# The association between the non-high-density lipoprotein cholesterol to high-density lipoprotein cholesterol ratio and the risk of osteoporosis among U.S. adults: analysis of NHANES data

**DOI:** 10.1186/s12944-024-02152-7

**Published:** 2024-06-03

**Authors:** Jinzhou Wang, Shanshan Li, Hongyu Pu, Jiangtao He

**Affiliations:** 1 The Department of Orthopaedic Surgery, the Affiliated Hospital of North Sichuan Medical College, Nanchong, Sichuan Province 637000 China; 2 Department of Hepatobiliary Surgery, First Affiliated Hospital of Chongqing Medical University, Chongqing, 400000 China; 3 The Department of Orthopaedic Surgery, Fushun People’s Hospital, Zigong, Sichuan Province 643000 China

**Keywords:** NHHR, Bone mineral density, Osteoporosis, NHANES

## Abstract

**Background:**

Osteoporosis and atherosclerosis frequently afflict older adults, and recent insights suggest a deeper connection between these conditions that surpasses mere aging effects. The ratio of non-high-density to high-density lipoprotein cholesterol (NHHR) has emerged as a novel lipid marker for evaluating the risk of cardiovascular diseases. Nonetheless, investigations into the correlation of the NHHR with the risk of developing osteoporosis remain unexplored.

**Methods:**

We collected NHHR and bone mineral density (BMD) data from 11,024 National Health and Nutrition Examination Survey (NHANES) participants between 2011 and 2018. Multivariate linear regression was employed to examine the correlation between BMD and NHHR. Smooth curves were employed to deal with the nonlinearity. To further account for the nonlinear link, we used a two-part linear regression model. The threshold effects were estimated using two components of a linear regression model. Subgroup and sensitivity analyses were carried out to ascertain the stability of the findings.

**Results:**

We discovered a negative relationship between the NHHR and lumbar spine BMD in all three models. An L-shaped curvilinear association existed between the NHHR and lumbar spine BMD, with a key inflection point of 6.91. The fully adjusted model showed that the BMD of the lumbar spine fell by 0.03 g/cm^2^ in those who were in the fourth quartile as opposed to the lowest quartile. The sensitivity analysis using unweighted logistic analysis verified the stability of the results. In addition, BMD in the nondiabetic group was more significantly affected by the negative effect of the NHHR in the subgroup analysis.

**Conclusions:**

According to this research, there appears to be a negative correlation between BMD and NHHR in US Adults. To clarify the precise physiological mechanisms by which the NHHR contributes to the onset of osteoporosis, more research is necessary.

## Introduction

Osteoporosis is a widespread bone disorder that affects the systemic skeletal system, and is characterized by diminished bone density and deterioration of the bone tissue microarchitecture. This condition results in heightened vulnerability to fractures, which are especially prevalent among individuals in their middle and later years [1]. Despite taking anti-osteoporosis drugs for a long time, one or more fragility fractures will occur in 25% of men and 44% of women over the age of 60 [2]. Such fractures can result in intense discomfort, impairment, and perhaps fatal consequences [3]. As the global population ages, the global incidence of osteoporosis has risen to 19.7%.This increased incidence is anticipated to have a substantial impact on both the medical and economic structures of society as a whole [4]. Like to osteoporosis, cardiovascular disease is a significant global health issue [5, 6], with dyslipidemia being a common risk factor for its onset [7]. Recent studies have shown that in addition to the degenerative processes related to aging, atherosclerosis and osteoporosis are common disease mechanisms that affect bone and blood vessel mineralization, and dyslipidemia is a key factor influencing bone health [8]. Therefore, scientists have paid close attention to the connection between dyslipidemia and osteoporosis. This study investigated the associations between the levels of typical lipoproteins, including high-density lipoprotein cholesterol (HDL-C) and low-density lipoprotein cholesterol (LDL-C), and osteoporosis. HDL cholesterol is viewed as a preventive element against cardiovascular disease due to its anti-atherogenic and antioxidant properties [9]. However, the connection between HDL-C and osteoporosis remains a subject of debate. A cross-sectional study found that HDL-C levels and bone density were positively correlated [10]. Conversely, another cross-sectional study indicated that high HDL-C levels increased the risk of osteoporosis, highlighting that this effect was more pronounced on women [11]. Views on the impact of non-HDL cholesterol on BMD are diverse and inconclusive. A strong negative relationship was found between BMD and LDL-C levels in a detailed study of the NHANES III cohort in Hong Kong and the U.S. [12]. In contrast, a cross-sectional study in China indicated a robust relationship between LDL levels and BMD among women [13]. In contrast, a study conducted in Greece with 591 postmenopausal women found no significant correlation between lipid characteristics and BMD [14]. The relative importance of HDL-C and non-HDL-C in osteoporosis remains debatable in light of the aforementioned research. The heterogeneity of various forms of non-HDL-C may differ, which may directly or indirectly influence the association between osteoporosis and non-HDL cholesterol. Furthermore, other lipoprotein ratios such as apoB100/apoAI are not part of standard test. Therefore, we require a unique comprehensive lipid parameter index. The NHHR is a modern and comprehensive metric for assessing atherogenic lipids. It has surpassed conventional lipid indicators in predicting cardiovascular disease risk [15]. The NHHR, a newly discovered type of lipoprotein ratio, accounts for the dual effect of HDL-C and non-HDL-C avoiding the limitations of previous lipid-only studies. The NHHR may provide new insights into monitoring the severity of osteoporosis, and because it only requires lipid profile measurements, the NHHR may become an efficient, convenient, and cost-effective metric for disease assessment. We used the NHANES 2011–2018 dataset to do a cross-sectional study as a consequence.

## Methods

### Participants in the NHANES study

**Fig. 1:**
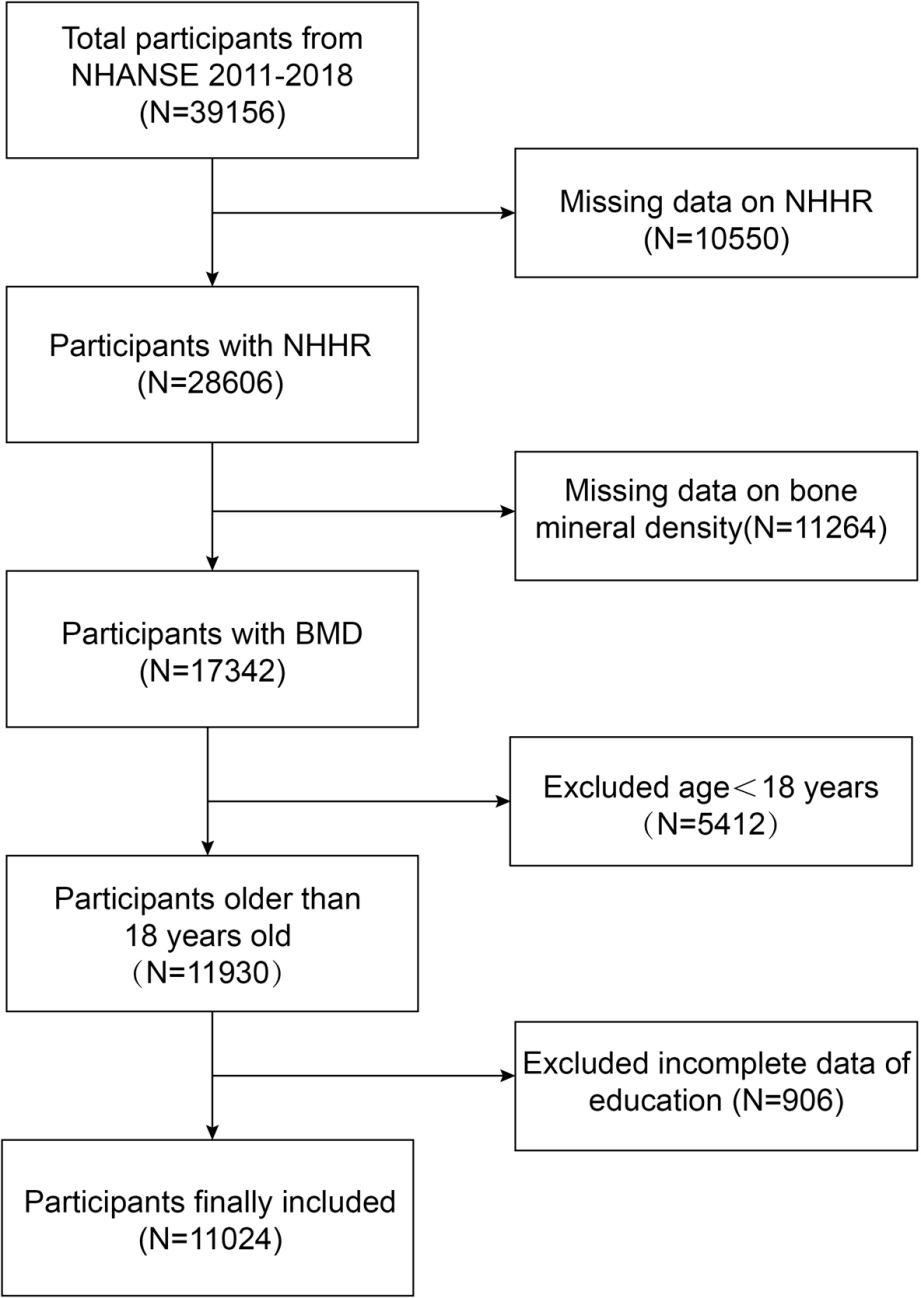
Process Map for Sample Collection from NHANES

From the 2011–2018 NHANES, we elected 39,156 individuals for our study. After screening, 11,024 participants were ultimately included in the present study (Fig. 1). We omitted people who lacked bone mineral data (11,264) or NHHR data (10,550) to investigate the relationship between the NHHR and osteoporosis. Moreover, individuals with incomplete education-related data (906), as well as those under the age of eighteen (5412), were excluded. The study protocol was approved by the National Center for Health Research Ethics Committee, and all participants provided signed informed permission. The National Center for Health Statistics website (http://www.cdc.gov/nchs/nhanes/) provided the population statistics. informed consent was granted in writing.

### BMD measurement and osteopenia/osteoporosis

Dual-energy X-ray bone densitometry was used to determine the bone density of the individuals. This method is widely respected and commonly utilized for evaluating bone density due to its favorable characteristics, including low radiation exposure, rapid processing, and simplicity [16]. A skilled radiology technician assessed the lumbar spine bone density of each patient enrolled in this study using dual-energy X-ray bone densitometry utilizing a Hologic QDR 4500A instrument. Based on the standards formulated by the National Bone Health Alliance Task Force, BMD readings ranging from -1 to -2.5 standard deviations (SDs) below the baseline suggest osteopenia. Furthermore, When BMD falls more than 2.5 SDs below the reference value, osteoporosis should be diagnosed [17]. Bone loss and osteoporosis occur at distinct anatomical locations in the lumbar region. Calculating the mean of measurements acquired from the initial to the fourth lumbar vertebra is required for the determination of lumbar spine bone mineral density [18].

### Measurement of the NHHR

NHHR was calculated based on the lipid levels of the subjects. The HDL-C level is subtracted from the total cholesterol level to determine the non-HDL-C level. The non-HDL-C level was divided by the HDL-C level to compute the NHHR. This study utilized a Roche Cobas 6000 analyzer and enzymatic assays to determine the concentrations of HDL-C and Total Cholesterol (TC).

### Covariates

Our analysis included other confounders known to potentially affect the association between the NHHR and osteoporosis. The covariates included sex, age, race, participation in moderate activities, marital status, poverty-to-income ratio (PIR), diabetes status, education level and history of smoking. Moderate activity is defined as moderate or low-intensity activity with a slight increase in respiration or heart rate, such as walking at a brisk pace or carrying light objects continuously for at least 10 min. Smoking status was defined as "Have you smoked at least 100 cigarettes in your lifetime?", "Has a doctor or other health professional ever told you that you have diabetes? " was the definition used to determine one's diabetes status. A person's BMI can be computed by dividing their height in meters squared by their weight in kilograms. This results in a kg/m^2^ figure [19].

### Statistical analysis

The statistical analyses incorporated NHANES sample weights to address the intricacies of multistage clustered surveys, in accordance with recommendations from the Centers for Disease Control and Prevention (CDC). While percentages were used to represent the categorical data, averages and standard deviations (SDs) were used to characterize the continuous variables. The NHHR data were normalized using a log2 transformation to guarantee an equitable distribution. The quartile rankings of the NHHR were used to divide the participants into four groups. Weighted Student's t-tests were used for continuous variables, and weighted chi-square tests were used for categorical variables to evaluate the baseline features of NHHR levels within these quartiles. Multiple linear regression analysis was used to investigate the relationship between the NHHR and BMD of the lumbar spine. This analysis required determining the beta coefficients and the 95% confidence intervals (CIs).The research was conducted utilizing three distinct models. The regression coefficients of the studies are displayed, with the lower quartile serving as the reference point. Model 1 remained unchanged and unaltered. Age, educational attainment, sex, and race were considered covariates during the adjustment process of Model 2. Model 3 exhibited the same attributes as Model 2, with the addition of supplementary modifications to account for PIR, BMI, moderate activity levels, diabetes, and smoking. We examined the nonlinear relationship between the NHHR and BMD using an estimating technique that combined a generalized additive model with a smoothing curve. Upon identifying nonlinearity, we adopted a recursive strategy to locate the inflection point within the connection between the BMD and NHHR. For a more detailed understanding of this nonlinear pattern, a biphasic linear regression model was applied around the identified inflection point. While the NHANES employs advanced sampling procedures to increase the representativeness and application of its findings, weighted and unweighted analyses may provide skewed results in some circumstances. We performed a sensitivity analysis with unweighted regression to revalidate our findings in this study. Subgroup analyses were then carried out to evaluate the data's consistency and dependability. The statistical analyses were carried out using the software programs PackageR and EmpowerStats, which are accessible at http://www.r-project.org and http://www.empowerstats.com, respectively. It was determined that statistical significance was indicated by a *P* value less than 0.05.

## Results

### Baseline characteristics

A group of 11,024 participants fulfilled the study's inclusion and exclusion criteria, with an average age of 39.50 ± 11.69 years. The participants were 52.12% male, 47.88% female, 61.43% non-Hispanic white, 10.30% Mexican American, 11.49% non-Hispanic black, and 9.52% from various other racial groups. Among all participants, the mean (SD) lumbar spine BMD and NHHR were 1.04 (0.15) g/cm^2^ and 7.05 (0.43), respectively. All clinical characteristics of the patients are listed by NHHR quartile in Table 1. There were noticeable variations in BMI, PIR, smoking status, education level, age, female, race, and marital status(*P* < 0.05). Individuals in the highest NHHR quartile were predominantly male, non-Hispanic white, and older than those in the lowest quartile. Furthermore, individuals with elevated NHHRs were characterized by a lower education and income, higher rates of smoking, increased BMI, decreased HDL-C levels, higher total cholesterol, and a lower BMD.

**Table 1:** Baseline characteristics of the study population according to the Non-HDL-C/HDL-C ratio

Variables	Q1	Q2	Q3	Q4	*P*-value
	*N* = 2697	*N* = 2812	*N* = 2694	*N* = 2821	
Age(years)	34.70 ± 11.72	38.81 ± 11.63	40.72 ± 11.24	43.55 ± 10.39	< 0.0001
Gender, n(%)					< 0.0001
Male	45.75	47.78	54.04	60.60	
Female	54.25	52.22	45.96	39.40	
Race, n(%)					< 0.0001
Mexican American	8.77	9.51	11.62	11.25	
Other Hispanic	6.80	7.13	7.21	7.88	
Non-Hispanic White	59.75	60.99	62.40	62.53	
Non-Hispanic Black	16.04	11.71	9.52	8.86	
Other Race	8.64	10.66	9.24	9.47	
Education Level, n(%)					0.0002
Less than high school	11.36	12.78	13.54	14.65	
High school	20.54	21.95	20.82	23.44	
More than high school	68.10	65.28	65.64	61.91	
Marital status, n(%)					< 0.0001
Yes	50.60	63.36	70.42	73.51	
No	49.40	36.64	29.58	26.49	
Diabetes, n(%)					0.1959
Yes	7.77	7.17	7.29	8.56	
No	92.23	92.83	92.71	91.44	
Smoking, n(%)					< 0.0001
Yes	28.08	30.32	32.61	37.57	
No	71.92	69.68	67.39	62.43	
Moderate activity, n(%)					0.1075
Yes	45.23	43.61	42.94	45.81	
No	54.77	56.39	57.06	54.19	
Body mass index(kg/m^2^)	26.84 ± 6.90	28.82 ± 6.99	30.03 ± 6.87	30.44 ± 6.07	< 0.0001
PIR	2.85 ± 1.69	2.90 ± 1.65	3.06 ± 1.66	3.00 ± 1.67	< 0.0001
Total Cholesterol (mg/dL, mean ± SD)	148.66 ± 20.33	176.24 ± 17.20	198.53 ± 15.73	240.41 ± 31.67	< 0.0001
HDL-Cholesterol (mg/dL, mean ± SD)	58.18 ± 16.83	54.60 ± 16.09	50.87 ± 14.26	46.91 ± 13.26	< 0.0001
Lumbar spine BMD (g/cm^2^, mean ± SD)	1.06 ± 0.15	1.05 ± 0.15	1.03 ± 0.15	1.01 ± 0.15	< 0.0001

Weighted characteristics of the study population based on Log2-NHHR quartiles,Mean ± SD for continuous variables: the *P* value was calculated by the weighted linear regression model,(%) for categorical variables: the *P* value was calculated by the weighted chi-square test

### Association between the NHHR and BMD

Table 2 presents a multivariate regression analysis that investigated the link between the log2-transformed NHHR and lumbar spine BMD. Initially, the unadjusted model indicated a negative relationship between BMD and NHHR (β = -0.04, 95% CI: -0.05 - -0.04, *P* < 0.0001). In Model 2, this link held statistical significance even after other factors were taken into account (β = -0.03, 95% CI: -0.04 - -0.02,* P* < 0.0001). Following a thorough covariate adjustment in Model 3, a consistent decrease in the lumbar spine BMD of 0.03 g/cm^2^ was observed for each unit increase in the NHHR, and the association was still significant (β = -0.03, 95% CI: -0.04 - -0.02, *P* < 0.0001). Within the context of the fully adjusted third model, it was observed that individuals in the highest NHHR quartile exhibited a BMD that was 0.03 g/cm^2^ lower than that of people who are in the bottom quartile (β = -0.03, 95% CI: -0.04 - -0.02, *P* < 0.0001).

**Table 2:** The association between log2-transformed NHHR and bone mineral density

Exposure	Model 1 [β (95% CI)]	Model 2 [β (95% CI)]	Model 3 [β (95% CI)]
Lumbar spine BMD(continuous)	-0.04 (-0.05, -0.04) < 0.0001	-0.03 (-0.04, -0.02) < 0.0001	-0.03 (-0.04, -0.02) < 0.0001
Lumbar spine BMD(quartile)			
Quartile 1	Reference	Reference	Reference
Quartile 2	-0.01 (-0.02, -0.00) 0.0123	-0.00 (-0.01, 0.01) 0.5118	-0.00 (-0.01, 0.01) 0.7990
Quartile 3	-0.03 (-0.03, -0.02) < 0.0001	-0.02 (-0.02, -0.01) 0.0002	-0.01 (-0.02, -0.01) 0.0008
Quartile 4	-0.05 (-0.05, -0.04) < 0.0001	-0.03 (-0.04, -0.02) < 0.0001	-0.03 (-0.04, -0.02) < 0.0001
*P* for trend	< 0.0001	< 0.000A1	< 0.0001
Stratified by diabetes			
Yes	-0.01 (-0.03, 0.01) 0.3669	-0.00 (-0.02, 0.02) 0.9683	0.00 (-0.02, 0.02) 0.9115
No	-0.05 (-0.06, -0.05) < 0.0001	-0.04 (-0.04, -0.03) < 0.0001	-0.03 (-0.04, -0.03) < 0.0001

Model 1: no covariates were adjusted

Model 2: age, gender, and race were adjusted

Model 3: age, gender, race, educational level, BMI, family income-to-poverty ratio, moderate activities, martial status, diabetes status, smoking, cancer were adjusted

### A nonlinear association between the NHHR and BMD

**Fig. 2:**
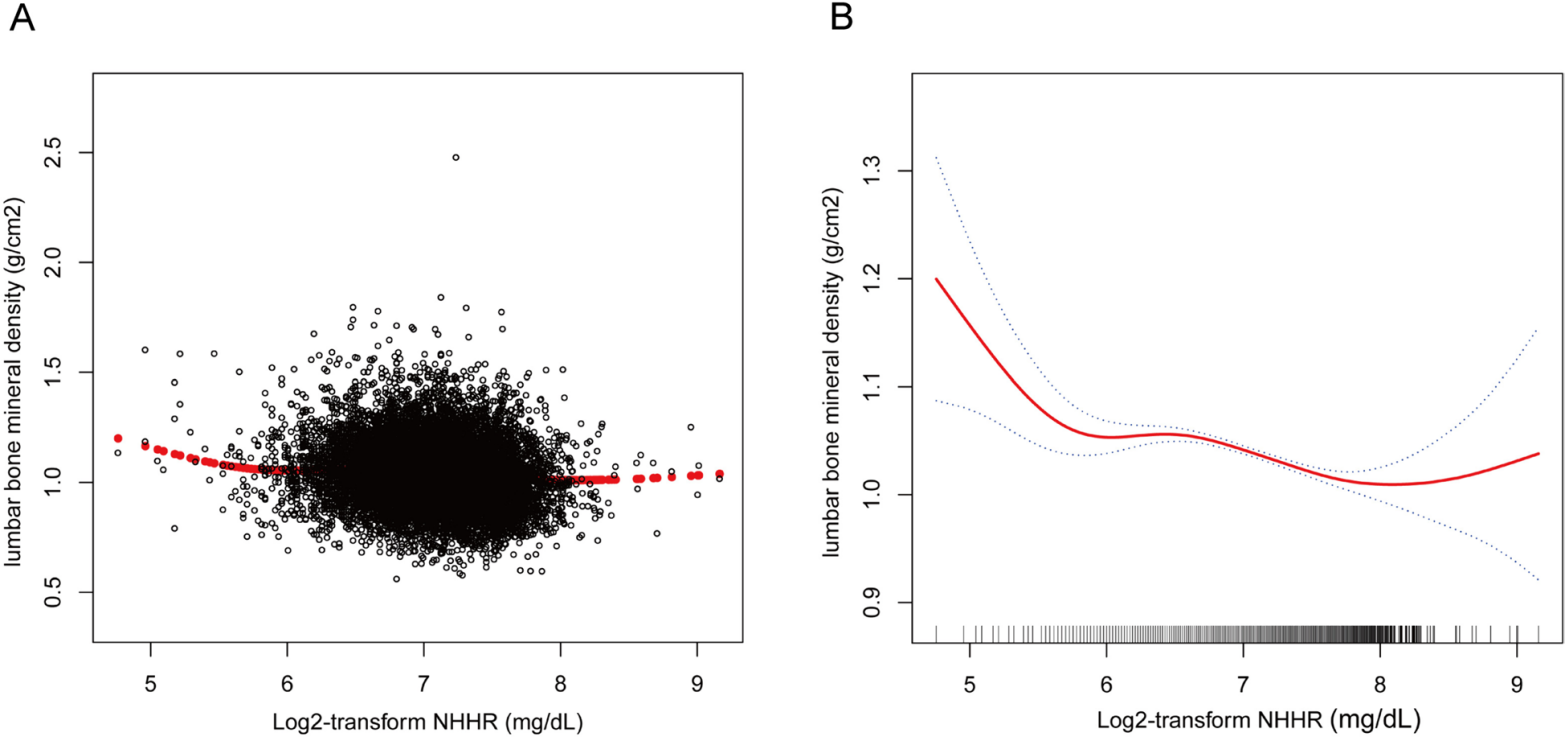
The association between NHHR and BMD. **A** Each black point represents a sample. **B** Blue bands represent the 95% confidence interval from the fit

This study employed smooth curve fitting and two-segment linear regression models to examine the nonlinear relationship between the NHHR and BMD of the lumbar spine. The findings underscored a nonlinear link, establishing a negative correlation between the NHHR and lumbar spine BMD, as illustrated in Fig. 2. Using a two-segment linear regression model, an L-shaped connection between the NHHR and BMD of the lumbar spine was identified, with a pivotal inflection point at 6.91, as outlined in Table 3.

**Table 3:** Threshold effect analysis of NHHR on lumbar spine bone mineral density

Lumbar Spine Bone Mineral Density	Adjusted β **(**95% CI)	*P*-value
Fitting by the standard linear model	-0.03 (-0.04, -0.02)	< 0.0001
Fitting by the two-piecewise linear model		
Inflection point	6.91	
NHHR < 6.91	-0.01 (-0.02, 0.01)	0.3305
NHHR > 6.91	-0.04 (-0.06, -0.03)	< 0.0001
Log likelihood ratio	< 0.001	

age, gender, race, educational level, marital status, BMI, family income-to-poverty ratio, moderate activities, diabetes status, smoking were adjusted

### Sensitivity analysis

Similarly, sensitivity analyses using unweighted logistic analyses showed that individuals with the highest quartile of the NHHR had a lower BMD than those with the lowest quartile of the NHHR, according to the different models, Model 1 (β = -0.05, 95% CI: -0.06 - -0.05, *P* < 0.0001), Model 2 (β = -0.03, 95% CI: -0.04 - -0.02, *P* < 0.0001), and Model 3 (β = -0.03, 95% CI: -0.04 - -0.03, *P* < 0.0001) (Table 4). The NHHR and BMD appear to consistently correlate negatively, according to these findings.

**Table 4:** Unweighted logistic regression analysis on the association between log2-transformed NHHR and BMD in sensitive analysis

Exposure	Model 1 [β (95% CI)]	Model 2 [β (95% CI)]	Model 3 [β (95% CI)]
Lumbar spine BMD(continuous)	-0.05 (-0.05, -0.04) < 0.0001	-0.03 (-0.04, -0.02) < 0.0001	-0.03 (-0.04, -0.02) < 0.0001
Lumbar spine BMD(quartile)			
Quartile 1	Reference	Reference	Reference
Quartile 2	-0.02 (-0.03, -0.01) < 0.0001	-0.01 (-0.02, 0.00) 0.0543	-0.01 (-0.02, 0.00) 0.0455
Quartile 3	-0.04 (-0.05, -0.03) < 0.0001	-0.02 (-0.03, -0.01) < 0.0001	-0.02 (-0.03, -0.01) < 0.0001
Quartile 4	-0.05 (-0.06, -0.05) < 0.0001	-0.03 (-0.04, -0.02) < 0.0001	-0.03 (-0.04, -0.03) < 0.0001
*P* for trend	< 0.0001	< 0.0001	< 0.0001

Model 1: no covariates were adjusted

Model 2: age, gender, and race were adjusted

Model 3: age, gender, race, educational level, BMI, family income-to-poverty ratio, moderate activities, martial status, diabetes status, smoking, cancer were adjusted

### Subgroup analysis

Further subgroup analyses were performed for associations in different population settings, including race, sex, education, smoking status, daily activities, and diabetes status. After adjusting for confounding factors, the effect size of each subgroup remained relatively stable, as shown in Table 5. No significant effects of race, sex, education, smoking status, or daily activities on the interaction test were observed. According to subgroup analyses based on the diabetes status, the BMD of the nondiabetic group was more significantly negatively affected by the NHHR. (β = -0.03, 95% CI: -0.04--0.03, *P* < 0.0001), However, in models relevant to diabetes, the association did not achieve statistical significance.

**Table 5:** Subgroup analysis of the association between Log2-NHHR and Bone mineral density

Subgroup	Lumbar BMD [β (95% CI)]	*P* for interaction
Gender		0.0504
Male	-0.04(-0.05, -0.03)	
Female	-0.02(-0.03, -0.01)	
Race/ethnicity		0.7253
Mexican American	-0.02(-0.04, -0.00)	
Other Hispanic	-0.03(-0.05, -0.01)	
Non-Hispanic White	-0.04(-0.05, -0.02)	
Non-Hispanic Black	-0.03(-0.04, -0.01)	
Other Race	-0.03(-0.05, -0.02)	
Education level		0.7220
Less than high school	-0.03(-0.04, -0.01)	
High school	-0.03(-0.04, -0.01)	
More than high school	-0.03(-0.04, -0.02)	
Moderate activity		0.2302
Yes	-0.04(-0.05, -0.03)	
No	-0.03(-0.04, -0.02)	
Diabetes status		0.0016
Yes	0.00(-0.02, 0.02)	
No	-0.03(-0.04, -0.03)	
Smoking		0.6319
Yes	-0.03(-0.04, -0.02)	
No	-0.03(-0.04, -0.02)	

## Discussion

For the first time, this study, which is based on a nationally representative survey in the U.S., demonstrated a adverse relationship between BMD and a greater NHHR. Even after taking into consideration every element in the category model, the adverse link between the NHHR and lumbar spine BMD remained significant. Furthermore, our multiple linear regression analysis suggested the existence of a potential nonlinear inverse relationship between the NHHR and BMD. Further threshold analyses revealed that the NHHR and lumbar spine bone density inflection point was 6.91, and that when the NHHR was greater than 6.91, the lumbar spine bone density decreased by 4% for each unit increase in the NHHR.

Osteoporosis and atherosclerosis are common in elderly people and result in substantial illness and death. Osteoporosis and atherosclerosis are increasingly believed to be biologically connected, and are not associated only with aging [20]. The new lipid ratio known as the NHHR is used to measure the degree of atherosclerosis. Although the significance of the NHHR in osteoporosis has not been investigated in any prior research, much discussion regarding the connection between HDL-C and LDL-C and osteoporosis has been documented. In a cohort study, a notable inverse correlation was detected between BMD and LDL-C levels in postmenopausal women [21]. Furthermore, a cross-sectional study including 4,441 individuals in their youth and middle years found that low-density lipoprotein (LDL) cholesterol significantly exacerbated osteoporosis [22]. In a cross-sectional analysis, a notable link was observed between the levels of HDL-C in the bloodstream and BMD among women who had gone through menopause [23]. In another cross-sectional investigation, the authors analyzed a sample of 20- to 59-year-olds and discovered that HDL-C levels were strongly and positively linked to lumbar spine BMD [24]. The findings of these previous studies indirectly validate our results. Furthermore, some evidence is available to corroborate our conclusions from animal research. Studies on animals have revealed that adult rats on a high-fat diet over an extended period of time have elevated levels of both total cholesterol and LDL-C, along with a significant decline in bone density [25]. Another study using animals revealed that mice lacking APOA1, a crucial molecule that controls the formation of HDL, had considerably lower bone densities than did mice with normal levels of this protein [26]. However, multiple researches have shown conflicting outcomes. In a MIDUS study of 440 participants the authors found that elevated blood levels of HDC-L were associated with a lower bone density [27], Another cross-sectional investigation of teenagers purportedly found a negative relationship between male adolescents' total BMD and HDL-C levels [28]. Furthermore, the authors of a cross-sectional study with 13,592 participants reported no associations between BMD and total cholesterol, LDL, or HDL levels in fully adjusted models [29]. Variations in participants and osteoporosis evaluations may impact the interpretation of the contentious research mentioned above. Thus, our research employs a novel lipid metric to enhance comprehension of the linkage between lipid levels and the risk of osteoporosis.

Understanding the underlying pathways linking lipid profiles and osteoporosis might help us in our research endeavors. Cholesterol is one of the most important components of osteoblasts and is directly involved in the construction and maintenance of cell membranes. Elevated cholesterol levels can disrupt many pathways involved in bone development, such as the Wnt and bone morphogenetic protein (BMP)/transforming growth factor β (TGF-β) pathways. Additionally, cholesterol has the capacity to inhibit the expression of BMP-2 and CBFAL and reduce ALP and collagen 1A levels in osteoblasts, thereby hindering osteoblast differentiation and promoting osteoclastogenesis. These changes impede the bone formation process and contribute to bone loss [30, 31]. Additionally, some studies have indicated that the main regulatory factors for adipocyte differentiation in bone marrow adipose tissue are PPARγ and CEBPa. The deficiency of ApoA1, the primary protein component of HDL-C, (corresponding to a reduction in high-density lipoprotein levels), may enhance the expression of Cebpa and PPARγ, thereby causing changes in the population of bone precursor cells, increasing adipocyte formation, and reducing osteoblast production [32]. Through its regulation of certain cell signaling pathways, including the tyrosine kinase receptor (RTK) system and the phosphatidylinositol-3-kinase (PI3K)/protein kinase B pathway, HDL contributes to signaling events that impact the development, specialization, and bone-forming activity of osteoblasts [33]. While oxidized LDL cholesterol significantly influences the osteoconversion process, It is important to recognize that inflammatory bioactive lipids play a part in bone turnover [34]. A study has shown that low-density lipoprotein oxidation products can cause bone turnover due to their ability to induce progenitor bone marrow stromal cells to grow in a lipogenic rather than bone-derived direction [35]. OxLDL has also been demonstrated to impede inorganic phosphate (Pi) signaling and degrade Pi-induced osteoblast development by causing oxidative stress [36]. Hyperlipidemia can potentially instigate enduring inflammation in the body, promoting the secretion of inflammatory agents like tumor necrosis factor-α (TNF-α) and interleukin-6 (IL-6) [37]. These substances have the potential to affect normal bone metabolism because osteoblast differentiation and activation are tightly controlled by the Wnt/β-catenin signaling axis. Extracellular Wnt must bind to a particular complex composed of LDL receptor related proteins (LRPs) and extracellular structural domains 5 and 6 of members of the frizzled receptor family (FZDs) to activate this axis [38]. Inflammatory factors (IL6, TNFα, etc.) can stimulate secretory frizzled-related protein (sFRP), Dickkopf, and sclerostin, thereby inhibiting Wnt—lrp—fzd assembly and preventing the downstream effects of the Wnt/β-catenin pathway on osteoblasts [39], which leads to inflammation-related bone loss. Statins act as blockers of hydroxymethylglutaryl coenzyme A (HMG-CoA) reductase and are able to reduce cholesterol synthesis, significantly lowering LDL cholesterol and Apo-B levels, in turn lowering serum triglyceride levels, and stabilizing and reversing plaques, thus, they are now becoming a fundamental drug in the fight against atherosclerosis and reduce the risk of cardiovascular disease [40, 41]. Research investigations and medical evidence indicate possible positive impacts on bone metabolism, because of its osteogenic properties [42, 43] and its ability to inhibit osteoclast activity [44].

## Strengths and limitations

This study has a number of noteworthy research strengths. First, the enormous sample size in this study reduces the influence of chance factors on the findings and increases the reliability of the conclusions. Second, previous studies on lipids and osteoporosis have primarily focused on independent lipid indices. This study is the first to use a composite Non-HDL-C/HDL-C ratio, confirming a substantial contact between the NHHR and BMD, which improves the accuracy the of clinical prediction. This study inevitably has several shortcomings. First, this study included only a sample of adult Americans; as a result, its findings may not be as applicable to other nations or ethnic groups, and further validation in a larger population is required. Second, the lumbar spine (L1-4) is a frequent location for BMD assessments, nevertheless, degenerative alterations in the spine can influence lumbar BMD. The lumbar DXA test may overestimate lumbar BMD, yielding false-negative findings [45, 46]. In recent years, some research has discovered that forearm BMD measurements might be a viable alternative approach. Forearm DXA is more accurate than lumbar DXA in postmenopausal women and those with spinal degeneration [47]. As a result, such an alternate approach might be used to quantify BMD in future investigations. Finally, although we considered many potential variables, the possibility of other factors that could influence the correlation between osteoporosis and the NHHR cannot be completely ruled out.

## Conclusion

The current study found that elevated NHHRs affect bone loss in the U.S. population, highlighting a potential clinical link between lipid profiles and bone metabolism. For individuals who are at high risk of developing osteoporosis, the current study offers helpful data in favor of primary osteoporosis prevention. Moreover, in clinical practice, health care professionals can incorporate the NHHR into clinical risk assessments of osteoporosis and provide early intervention for those with elevated NHHRs to reduce the risk of osteoporosis, in addition to helping to improve patient management and clinical decision-making by health care professionals for patients with osteoporosis, and to improve the effectiveness of anti-osteoporosis treatment.

## Data Availability

Data from the survey is publicly available online at http://www.cdc.gov/nchs/nhanes for data users worldwide.

## Abbreviations

BMI: Body mass index
BMD: Bone mineral density
NHANES: National Health and Nutrition Examination Survey
PIR: Poverty-to-income ratio
NHHR: The ratio of Non-HDL-C to HDL-C
HDL-C: High-density lipoprotein cholesterol
LDL-C: Low-density lipoprotein cholesterol
